# Global Reach of Conceptual Models Used in Ageism and Dementia Studies: A Scoping Review

**DOI:** 10.1093/geroni/igaa057.3185

**Published:** 2020-12-16

**Authors:** Atiqur Rahman, Lars-Christer Hyden

**Affiliations:** Linkoping University, Norrköping, Ostergotlands Lan, Sweden

## Abstract

Older adults are discriminated against at different social levels- called ageism and the people living with dementia (PlwD) not only face ageism due to being elder but also encounter prejudices, negative attitudes and discrimination because they live with dementia. The purpose of this review is to highlight the different conceptual and methodological approaches used and the groups being investigated as well as the findings from studies in the combined research field of ageism and dementia. Five scientific databases were used within a timeframe between 2009 and 2018. A total number of 98 articles (quantitative, qualitative, and mixed methods) were included and analyzed based on (i) the conceptual frameworks, (ii) methodological approaches, (iii) groups studied, and (iv) findings. The result emerged six themes (i) stereotypes and attitudes in the general population; (ii) stereotypes and attitudes of themselves; (iii) stereotypes and attitudes among staff; (iv) stereotypes and attitudes as part of culture; (v) stereotypes and attitudes of family members; and (vi) miscellaneous. The principal findings identified lower education, lack of knowledge and less information about dementia as the main contributing factors to manifest stereotypes, attitudes, and ageism in society toward PlwD that leads to stigma. Moreover, this review found few studies were based on explicit conceptual frameworks but rather on descriptive understandings of relations between attitudes. There is a domination of the quantitative approach where the target populations were mostly from Western regions. The review suggests that a more conceptually coherent approach would allow for a better cumulative building of knowledge.

